# CXCR4 and CCR5 shRNA transgenic CD34+ cell derived macrophages are functionally normal and resist HIV-1 infection

**DOI:** 10.1186/1742-4690-2-53

**Published:** 2005-08-18

**Authors:** Joseph Anderson, Ramesh Akkina

**Affiliations:** 1 Dept. Microbiology, Immunology and Pathology, Colorado State University, Fort Collins, Colorado 80523, USA

## Abstract

**Background:**

Stable simultaneous knock down of the HIV-1 coreceptors CCR5 and CXCR4 is a promising strategy to protect cells from both R5 macrophage tropic and X4 T cell tropic as well as dual tropic viral infections. The potency of shRNAs in targeted gene silencing qualifies them as powerful tools for long term HIV gene therapy. Our previous work with a bispecific lentiviral vector containing CXCR4 and CCR5 shRNAs showed efficacy in down regulating both coreceptors and conferring viral resistance to both X4 and R5-tropic strains of HIV-1 in cultured cell lines. To extend these results to a stem cell gene therapy setting, here we show transduction of primary CD34+ hematopoietic progenitor cells to derive normal end stage cells that are resistant to HIV-1 infection.

**Results:**

The bispecific XHR lentiviral vector harboring CXCR4 and CCR5 shRNA expression cassettes was efficient in transducing CD34+ cells. The transduced cells gave rise to morphologically normal transgenic macrophages when cultured in cytokine media. There was a marked down regulation of both coreceptors in the stably transduced macrophages which showed resistance to both R5 and X4 HIV-1 strains upon *in vitro *challenge. Since off target effects by some shRNAs may have adverse effects on transgenic cells, the stably transduced macrophages were further analyzed to determine if they are phenotypically and functionally normal. FACS evaluation showed normal levels of the characteristic surface markers CD14, CD4, MHC class II, and B7.1. Phagocytic functions were also normal. The transgenic macrophages demonstrated normal abilities in up-regulating the costimulatory molecule B7.1 upon LPS stimulation. Furthermore, IL-1 and TNFα cytokine secretion in response to LPS stimulation was also normal. Thus, the transgenic macrophages appear to be phenotypically and functionally normal.

**Conclusion:**

These studies have demonstrated for the first time that a bispecific lentiviral vector could be used to stably deliver shRNAs targeted to both CCR5 and CXCR4 coreceptors into CD34+ hematopoietic progenitor cells and derive transgenic macrophages. Transgenic macrophages with down regulated coreceptors were resistant to both R5 and X4 tropic HIV-1 infections. The differentiated cells were also phenotypically and functionally normal indicating no adverse effects of shRNAs on lineage specific differentiation of stem cells. It is now possible to construct gene therapeutic lentiviral vectors incorporating multiple shRNAs targeted to cellular molecules that aid in HIV-1 infection. Use of these vectors in a stem cell setting shows great promise for sustained HIV/AIDS gene therapy.

## Background

Gene therapy approaches using the strategy of intracellular immunization hold considerable promise towards controlling HIV infection. Previous attempts with anti-HIV molecules that employed RNA decoys, transdominant proteins, and ribozymes were promising towards developing novel therapies [1-12]. With the recent discovery of RNA interference (RNAi), a new and more powerful tool has become available to add to the growing anti-HIV arsenal. The phenomenon of RNA interference has proven to be highly potent in post-transcriptional gene silencing [13-15]. Mediated by sequence specific small-interfering RNAs (siRNAs), RNAi can effectively down regulate the expression of either viral or cellular RNA targets by selective degradation of homologous mRNAs [16]. The mechanism of mRNA degradation involves an endonuclease present in the RNA-induced silencing complex (RISC) which is guided by the antisense component of the siRNA for target recognition [13,14]. A number of reports have shown that delivery of siRNAs by transfection of presynthesized siRNAs or plasmids encoding siRNAs into cultured cells can effectively inhibit HIV-1 infections [17-26]. However, due to the transient nature of transfected nucleic acid, the antiviral effects are only temporary. For HIV gene therapy strategies to succeed long range, it is necessary that siRNA coding transgenes be maintained and expressed long term in a virus susceptible target cell. In this regard, lentiviral vectors have proven to be highly effective in high efficiency gene transduction and sustained gene expression [27-32].

A number of studies using siRNAs have targeted HIV genes as well as the cellular molecules critical for HIV entry, namely CD4, CXCR4 and CCR5 [18,19,21,23,24,33-37]. SiRNAs targeting HIV genes alone will not be sufficient to ward off chronic infection due to the high possibility of generating escape mutants [38,39]. Therefore by targeting host cellular genes critical for viral entry and/or replication, a more sustained efficacy of antiviral effects may be obtained. As a critical player in immunological function, CD4 is physiologically indispensable. The chemokine receptors CXCR4 and CCR5 also play critical roles as coreceptors for viral entry during infection with T cell tropic X4 and macrophage tropic R5 HIV-1 viral strains respectively [40,41]. Their sustained knock down may prove to be more efficacious for long range siRNA therapy.

Since both R5 and X4-tropic viral strains are involved in disease pathogenesis, it is important to consider both coreceptors when developing effective therapeutics. In a segment of the human population, a naturally occurring 32-bp deletion in the CCR5 gene results in the loss of coreceptor function thus conferring significant resistance to HIV infection [42-44]. Homozygous or heterozygous individuals with this mutation remain physiologically normal. With regard to the CXCR4 coreceptor, it was found to be dispensable for T cell development and maturation in murine studies [45].

Based on this rationale, recent work with synthetic siRNAs demonstrated that down regulating either CXCR4 or CCR5 will protect cells from X4 or R5 HIV-1 strains, respectively, at the level of viral entry [18,19,21,23,24,33-37]. Stable expression of an anti-CCR5 siRNA was also achieved using a lentiviral vector. However, down regulating CCR5 alone in the face of an HIV-1 infection is insufficient [34]. Therefore, we recently demonstrated that synthetic bispecific combinatorial constructs as well as a bispecific lentiviral vector targeting both CXCR4 and CCR5 showed efficacy in inhibiting HIV-1 infections in cell culture lines [24,37]. In translating these findings into a stem cell gene therapy setting, this bispecific lentiviral vector was used in the present studies to generate shRNA expressing transgenic macrophages.

Macrophages, along with T cells, are major cell targets of HIV infections. Programming these cells to express shRNAs targeted to the essential coreceptors, CXCR4 and CCR5, could confer resistance to HIV infection. Macrophages also have a significant role in immune system functions as antigen presenting cells and as major effector cells in inflammation. Therefore, protecting macrophages from HIV infection is important in maintaining immune system homeostasis. Since shRNAs can have possible off target effects thus dysregulating cellular physiology, transgenic macrophages also need to be assessed for proper functionality [46]. Here we show that CD34+ hematopoietic progenitor cell derived macrophages expressing shRNAs targeting CXCR4 and CCR5 are functionally normal and resist infection to both X4 and R5-tropic strains of HIV-1.

## Results

### Lentiviral vector transduction of CD34+ cells with CXCR4 and CCR5 shRNAs and derivation of mature macrophages

**Figure 1:**
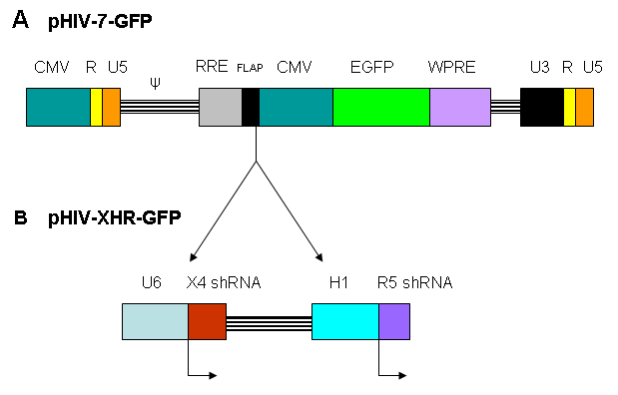
**Bispecific lentiviral vector (XHR) encoding anti-CXCR4 and CCR5 shRNAs**: A) Control transfer vector pHIV-7-GFP encoding a CMV promoter driven EGFP reporter gene. B) To derive the bispecific vector pHIV-XHR-GFP, a U6 promoter driven short hairpin CXCR4 shRNA cassette was cloned into the *BamH*I site upstream of the CMV-EGFP cassette. The H1-CCR5 shRNA cassette was inserted into an *Mlu*I site downstream to the U6-CXCR4 shRNA cassette.

A bispecific lentiviral vector XHR, coding for an shRNA targeting CXCR4 driven by a U6 promoter and a CCR5 shRNA under the control of an H1 promoter was designed as previously described (Fig. 1) [37]. This vector also contains an EGFP reporter gene downstream from the shRNA cassettes. CD34+ hematopoietic progenitor cells were transduced with either control GFP or XHR vectors. Cells were then sorted for EGFP and driven towards a myeloid lineage in semi-solid methyl cellulose cytokine media to generate transgenic macrophages. No significant differences were found in the levels of macrophages obtained when compared between the control GFP vector and XHR vector transduced cells or control non-transduced CD34+ cells. The morphology of the transgenic macrophages also appeared normal (data not shown).

### Down regulation of HIV-1 coreceptors CXCR4 and CCR5 in transgenic macrophages

**Figure 2:**
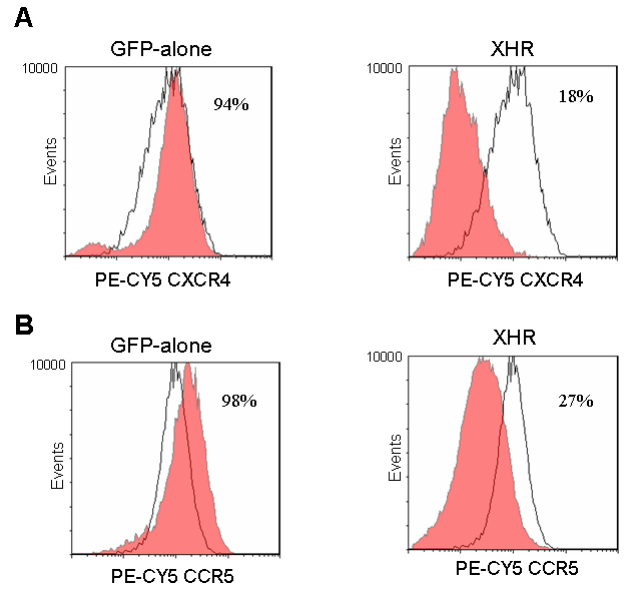
**Down regulation of the coreceptors CXCR4 and CCR5 in XHR transgenic macrophages**: GFP-alone and XHR transduced CD34+ derived macrophages were labeled with PE-CY5 conjugated antibodies specific for CXCR4 (A) and CCR5 (B) and analyzed by FACS. Control, nontransduced macrophages are shown superimposed as unshaded areas.

CD34+ derived macrophages normally express both major HIV-1 coreceptors, CXCR4 and CCR5, albeit a lower level of CXCR4. In XHR transduced cells FACS analysis showed an 82% decrease in CXCR4 expression. GFP-alone control vector transduced cells and non-transduced cells displayed normal levels of CXCR4 expression (94%) (Fig. 2A). Similar analysis for CCR5 expression showed a 73% decrease in XHR transduced macrophages with normal levels seen in GFP-alone vector transduced cells similar to non-transduced cells (98%) (Fig. 2B). Thus, stably transduced macrophages exhibited significant down regulation of both the coreceptors CXCR4 and CCR5 due to shRNA targeting.

### XHR transgenic macrophages resist HIV-1 challenge

**Figure 3:**
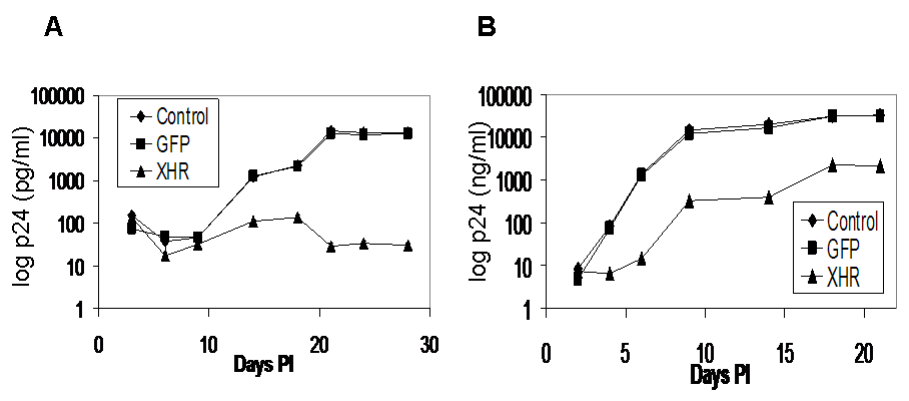
**HIV-1 resistance of XHR transgenic macrophages**: Control nontransduced (◆), GFP-alone (■), and XHR (▲) transduced CD34+ derived macrophages were challenged with (A) X4-tropic NL4-3 and (B) R5-tropic BaL-1 strains of HIV-1. p24 ELISAs were performed on culture supernatants taken at various time points post-infection. Experiments were performed in triplicate.

To determine if down regulation of CXCR4 and CCR5 coreceptors conferred viral resistance, transduced macrophages were challenged with X4-tropic (NL4-3) and R5-tropic (BaL-1) strains of HIV-1. Antigen ELISAs to detect viral p24 in culture supernatants were performed on various days post-infection. Over a 2-log reduction in viral yield was seen in XHR transduced macrophages challenged with X4-tropic HIV-1 as compared to control cells (Fig. 3A). In BaL-1 challenge experiments, there was over a 1-log reduction in viral titer in XHR transduced macrophages compared to control cells (Fig. 3B). Thus stable coreceptor down regulation by siRNAs resulted in marked protection of transgenic macrophages against viral challenge.

### Transgenic macrophages display characteristic phenotypic cell surface markers

**Figure 4:**
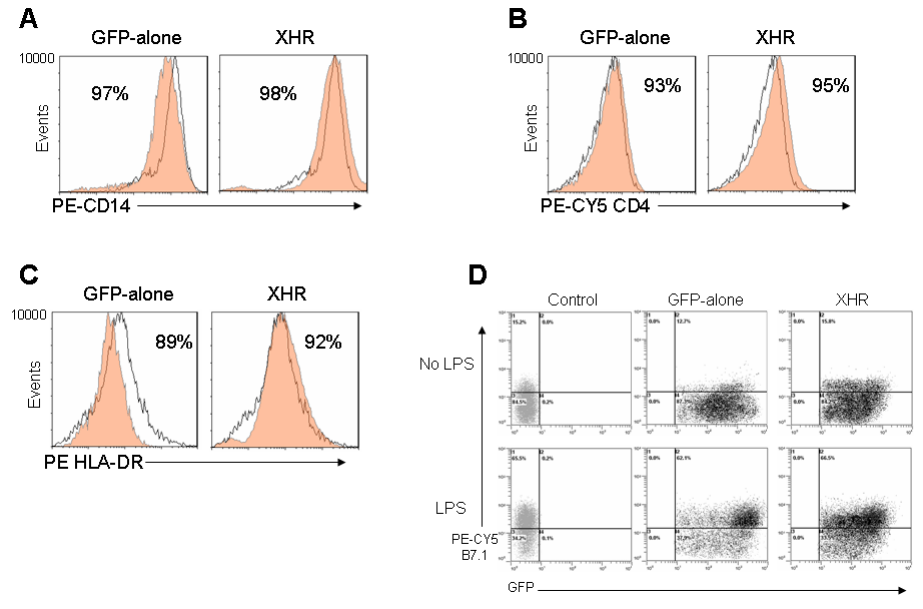
**Transgenic macrophages display normal cell surface markers**: GFP-alone and XHR transduced CD34+ derived macrophages were labeled with antibodies specific for (A) CD14, (B) CD4, and (C) HLA-DR and analyzed by FACS. Control, nontransduced macrophages are shown superimposed as unshaded areas. (D) B7.1 upregulation of transgenic macrophages stimulated with LPS. Twenty-four hours post-stimulation, macrophages were labeled with a PE-CY5 conjugated anti-B7.1 antibody and analyzed by FACS. B7.1 upregulation data are representative of triplicate experiments.

Macrophages are critical players in the immune system and also participate in the inflammatory response. Recent work demonstrated possible off target effects of some siRNAs [46]. Such effects may disrupt the phenotypic properties of macrophages or alternatively, may interfere with their normal function. Therefore, transgenic macrophages were subjected to phenotypic analyses to assess their characteristic cell surface markers by FACS. Levels of the monocyte/macrophage marker CD14 in XHR macrophages were found to be similar to GFP-alone transduced or nontransduced cells (98% and 97% respectively) (Fig. 4A). Similarly the levels of CD4, a primary HIV-1 receptor, were found at comparable levels for XHR and GFP-alone transduced macrophages at 95% and 93% respectively, coinciding with levels in nontransduced cells (Fig. 4B). The antigen presenting cell surface specific marker, HLA-DR (MHC II) present on macrophages is critical for presenting antigen to CD4+ T cells. A second co-stimulatory molecule B7.1 needed to activate T cells is present at low levels on normal macrophages. Its expression is elevated upon activation with certain stimuli such as LPS. Our evaluation showed that XHR transgenic macrophages displayed similar levels of HLA-DR (92%) when compared to GFP-alone (89%) or with non transduced macrophages (Fig. 4C). The levels of the costimulatory molecule B7.1 were found to be normal at ~15% without stimulation. The transgenic macrophages also displayed capacity to upregulate B7.1 (65%) after LPS stimulation similar to that seen with vector alone and non-transduced control cells (Fig. 4D).

### Transgenic macrophages are functionally normal

**Figure 5:**
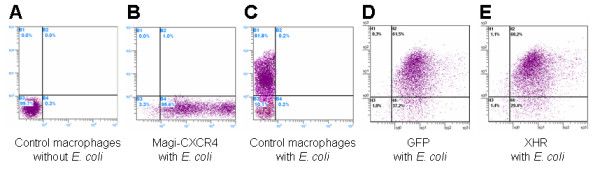
**Phagocytosis of fluorescently labeled E.coli by CD34+ derived macrophages**: E. coli Bioparticles^® ^were added directly to the cultured macrophages along with 5 μg/ml LPS. Twenty four hours post-stimulation, cells were analyzed by FACS. (A) Control macrophages without Bioparticles^®^. Panels B-E show plots of cells incubated with Bioparticles^® ^(B) Transduced Magi-CXCR4 (non-phagocytic cell culture), (C) nontransduced, (D) GFP-alone, and (E) XHR macrophages. These data are representative of triplicate experiments.

As stable expression of some shRNAs could have possible off-target global effects leading to disruption of normal cellular functions, we performed functional assays on transgenic macrophages to evaluate this possibilty. A typical function of macrophages is phagocytosis of foreign material and presentation of antigenic peptides. To determine if XHR transgenic macrophages retained the phagocytic function, they were presented with fluorescently labeled *E. coli *(Bioparticles^®^). Foreign cell uptake was measured by FACS. In comparing non-transduced, GFP-alone transduced, and XHR transduced macrophages, no significant differences in the phagocytic capacity were found between the transgenic macrophages and the vector alone transduced or non-transduced cells. Based on fluorecscence levels, XHR macrophage phagocytosis was quantified at 68.2% (Fig. 5E) compared to non transduced and GFP-alone cells at 63.5% and 61.5%, respectively (Fig. 5C and 5D). Transduced Magi-CXCR4 cells, serving as non-phagocytic cell controls did not display any phagocytic activity (Fig. 5B).

**Figure 6:**
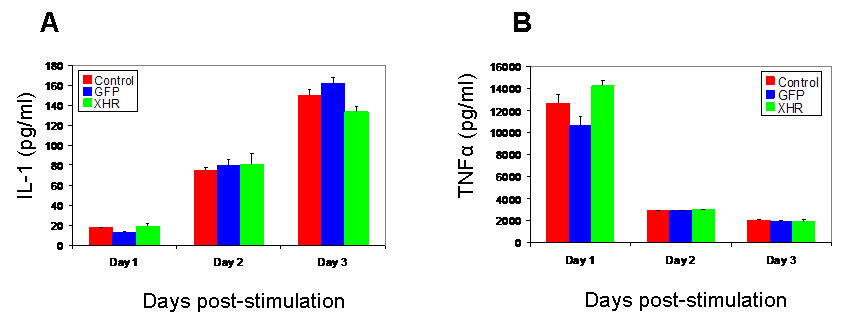
**XHR transgenic macrophages secrete normal levels of the cytokines IL-1 and TNFα**: Control nontransduced, GFP-alone, and XHR macrophages were stimulated with 5 μg/ml LPS. On days 1, 2, and 3 post-stimulation, supernatants were collected and assayed by ELISA for cytokine secretion of (A) IL-1 and (B) TNFα. Experiments were done in triplicate.

Due to their role in immunity and inflammatory response, macrophages secrete and respond to a number of important cytokines that include IL-1 and TNF-α. To determine if siRNA transgenic macrophages retained their functional capacity to secrete these cytokines at normal levels, they were stimulated with LPS. Levels of released cytokines were measured by ELISA. No significant differences were seen in levels of IL-1 and TNF-α cytokine secretion among the transgenic and control cell types (Fig. 6A and 6B). Basal levels of cytokine production were also detected without LPS stimulation with no differences seen between cell types (data not shown). Collectively the above data showed that coreceptor siRNA transgenic macrophages were phenotypically and functionally normal.

## Discussion

Down regulation of the major HIV-1 coreceptors CXCR4 and CCR5 in virus susceptible cells is a promising approach to prevent viral entry and establishment of productive infection. As noted above, targeting both coreceptors simultaneously will have the added advantage of protecting cells from both X4 and R5 tropic viruses as well as dual tropic strains. In the present studies we have shown that a bispecific lentiviral vector was effective in transducing the respective siRNAs targeted to these coreceptors into primary CD34+ hematopoietic progenitor cells which can give rise to all the blood cell lineages including macrophages, T cells, and dendritic cells.

Since siRNAs are new tools being used for genetic manipulation, it is necessary that they be systematically evaluated in a stem cell setting for their long range utility in protecting end stage differentiated cells such as macrophages. Recent studies have demonstrated that some siRNA constructs may have off target effects [46]. This may adversely affect cell differentiation pathways. Our results have demonstrated that mature macrophages could be derived from lentivirally transduced shRNAs targeting both CXCR4 and CCR5. No significant differences were found in the yields of macrophages from control non-transduced, control GFP-alone vector, and the bispecific shRNA vector transduced CD34+ cells when cultured in cytokine media permitting cell differentiation. This suggests that the respective shRNAs did not interfere with the lineage specific differentiation of gene transduced CD34+ cells into macrophages.

The transgenic macrophages showed significant down regulation of the respective targeted coreceptors CXCR4 and CCR5. Thus, differentiated cells retained functional shRNAs that were effective against their respective target mRNAs. When challenged with HIV-1 *in vitro *they showed marked resistance to infection with both X4 and R5 tropic viral strains. Most primary infections with HIV-1 are believed to be caused by R5 tropic HIV-1 as it is transmitted with relative ease with macrophages as the initial *in vivo *target. During disease progression, X4 tropic viruses are believed to emerge. However recent studies showed that primary X4 HIV-1 isolates could also infect macrophages obtained from human tissue establishing that initial infection of these cells *in vi*vo is not confined to R5 strains [51]. Therefore, protecting macrophages against both R5 and X4 tropic viruses is essential to prevent initial viral infection. Thus, the bispecific lentiviral vector harboring both CXCR4 and CCR5 shRNAs, described here, would be ideal in preventing HIV-1 infection at the cell entry stage.

A requirement for successful HIV-1 gene therapy is for transgenic virus resistant cells to be phenotypically and functionally normal to maintain and restore the body's immunological function. Accordingly, transgenic macrophages were evaluated to determine if they met these criteria. Although the levels of coreceptor expression diminished substantially as a result of shRNA targeting, phenotypic analyses of shRNA transgenic macrophages showed that they were otherwise phenotypically normal. This was shown by the comparable levels of CD14 and CD4 cell surface markers for both control cells and shRNA transgenic macrophages. Levels of the MHC class II molecule HLA-DR were also found to be normal. Upregulation of the costimulatory molecule B7.1 in response to LPS stimulation was comparable between shRNA transgenic and control vector containing cells. Furthermore, phagocytic functions were also found to be normal. To analyze the critical function of macrophages in secreting cytokines during the inflammatory response, the levels of IL-1 and TNF-α secretion were analyzed. Our results demonstrated that the expression of CXCR4 and CCR5 shRNAs and the subsequent downregulation of these chemokine receptors had no apparent effect on IL-1 or TNF-α secretion. These data collectively suggest that phenotypically and functionally normal macrophages could be obtained from CD34+ cells lentivirally transduced with CXCR4 and CCR5 shRNA constructs. These results establish for the first time that simultaneous knock down of both the chemokine receptors CXCR4 and CCR5 have no apparent adverse effects on macrophage differentiation, phenotype or function.

The above data showed the efficacy of this bispecific shRNA construct in deriving HIV-1 resistant macrophages *in vitro *in a stem cell setting. Further preclinical testing of this construct is needed *in vivo *to determine its suitability for use in the human. The SCID-hu mouse model that harbors a functional human thymus permits evaluation of vector transduced CD34+ cells to determine their capacity to give rise to mature T cells. The transgenic T lymphocytes so derived could be assessed for their functionality and viral resistance as we have shown previously [29]. Adverse effects are not expected by the stable knock down of CCR5 *in vivo *as it was previously documented in many studies that individuals harboring a 32 bp deletion in the CCR5 gene do not exhibit any immunological abnormalities [42-44]. However, stable CXCR4 knock down may have possible side effects in a stem cell setting due to its role in cell homing [52]. Therefore, a systematic evaluation of the CCR5 and CXCR4 bispecific construct *in vivo *in the SCID-hu mouse model is necessary to determine its efficacy and possible toxicity in differentiated T cells prior to its evaluation in human subjects. Such studies are currently underway.

## Conclusion

Stable simultaneous knock down of both the coreceptors CCR5 and CXCR4 is necessary to prevent HIV-1 infection at the entry level by both R5 and X4, as well as dual tropic viral strains. Our present studies have demonstrated for the first time that a bispecific lentiviral vector could be used to stably deliver shRNAs targeted to both CCR5 and CXCR4 coreceptors into CD34+ hematopoietic progenitor cells and derive transgenic macrophages. Stable down regulation of both the coreceptors was achieved in transgenic macrophages which displayed marked resistance to HIV-1 challenge in vitro. The siRNA expressing macrophages were also found to be phenotypically and functionally normal. It is now possible to construct gene therapeutic lentiviral vectors incorporating multiple siRNAs targeted to cellular molecules that aid in HIV-1 infection. Use of these vectors in a stem cell setting shows great promise for sustained HIV/AIDS gene therapy.

## Methods

### Generation of CXCR4 and CCR5 bispecific siRNA lentiviral vector XHR

A third-generation lentiviral vector system was used to produce the bispecific shRNA-expressing lentiviral vector [47]. The transfer vector pHIV-7-GFP was designed to contain an anti-CXCR4 shRNA cassette under the control of the Pol-III U6 promoter and an anti-CCR5 shRNA cassette under the control of the Pol-III H1 promoter, as previously described [37]. The anti-CXCR4 shRNA targets the CXCR4 transcript at nucleotides 3–23 and the anti-CCR5 shRNA targets the CCR5 transcript at nucleotides 13–31. A depiction of this bispecific lentiviral vector along with two important *cis*-acting elements is shown (Fig. 1). The two *cis*-acting elements, namely, the central DNA flap consisting of the cPPT and CTS (to facilitate the nuclear import of the viral preintegration complex) and the WPRE (to promote nuclear export of transcripts and/or increase the efficiency of polyadenylation of transcripts), are used to enhance the performance of the vector [47,48]. To generate lentiviral vectors, 293T cells, maintained in complete DMEM containing 10% FBS, were transfected with the plasmids pCHGP-2, pCMV-Rev, pCMV-VSVG, and the appropriate transfer vector, GFP-alone or XHR, using a calcium phosphate transfection kit (Sigma-Aldrich, St. Louis, MO). Cell culture supernatants were collected at 24, 36, 48, and 60 hours post-transfection, pooled, and concentrated by ultracentrifugation. Vector titers were then analyzed on 293T cells by FACS for EGFP expression. Concentrated vector titers ranged from 8.0 × 10^7 ^to 1.5 × 10^8 ^for XHR and GFP-alone vectors, respectively.

### Transduction of CD34+ hematopoietic stem cells and derivation of macrophages

CD34+ hematopoietic progenitor cells were purified from human fetal liver by selection with monoclonal antibody-conjugated immunomagnetic beads (Miltenyi Biotech, Auburn, CA)[8]. The purity of CD34+ cells was determined by FACS using a PE conjugated CD34+ antibody. The purity of cells was routinely >93% (data not shown). CD34+ cells were maintained in Iscove's modified Dulbecco's growth medium containing IL-3, IL-6, and stem cell factor (SCF) each at 10 ng/ml (R&D Systems, Minneapolis, MN) supplemented with 10% FBS. Lentiviral vector transductions were performed on 2 consecutive days at an m.o.i. of 30 in the presence of polybrene (4 ug/ml). Transduced cells were then sorted by FACS for EGFP expression and subsequently placed in semi-solid methylcellulose Methocult media (Stem Cell Technologies, Vancouver, BC, Canada) for 10–12 days to derive myeloid colonies. Total myeloid colonies were then pooled and cultured *in vitro *in DMEM supplemented with the cytokines M-CSF (25 ng/ml) and GM-CSF (25 ng/ml) (R&D Systems, Minneapolis, MN) for 4 days to derive mature macrophages.

### Phenotypic and functional analysis of transgenic macrophages

To determine if stem cell derived anti-coreceptor shRNA transgenic macrophages were otherwise phenotypically normal, analysis of macrophage cell surface markers was performed by FACS with respective conjugated antibodies, PE-CD14 (Caltag, Burlingame, CA), PE-HLA-DR, PE-CY5-CD4, PE-CY5-CXCR4, and PE-CY5-CCR5 (BD Biosciences, San Jose, CA).

Activated macrophages up-regulate the expression of B7.1 co-stimulatory molecules upon stimulation with various stimuli. Accordingly, control non-transduced, GFP-alone, and XHR vector transduced macrophages were stimulated with LPS (5 μg/ml) (Sigma-Aldrich, St. Louis, MO). Twenty-four hours post-stimulation, macrophages were stained with PE-CY5 conjugated anti-B7.1 antibody (BD Biosciences, San Jose, CA) and analyzed by FACS. FACS analyses were performed on the Beckman Coulter Epics XL using ADC software for analysis.

Macrophages play an important role in the immune system as phagocytes. To determine if XHR transgenic macrophages retained the ability to phagocytose foreign material, a phagocytosis assay utilizing tetramethylrhodamine fluorescently labeled *E. coli *Bioparticles^® ^(Invitrogen, Carlsbad, CA) were used. To the cell culture media, 5 ug/ml of LPS and 5 ug/ml of *E. coli *particles were added. Twenty-four hours post-addition, cells were analyzed by FACS. Transduced Magi-CXCR4, maintained as previously described [49,50], were used as a non-phagocytic cell control. Bioparticles^® ^were detected in the PE (FL2) channel for FACS analysis.

Transgenic macrophages were also analyzed for the secretion of two major cytokines, IL-1 and TNF-a. Macrophages were stimulated with 5 ug/ml of LPS. On days 1, 2, and 3 post-stimulation, cell culture supernatant samples were collected and analyzed by a Quantikine^® ^ELISA kit (R&D Systems, Minneapolis, MN). Non-stimulated supernatants were also analyzed for basal levels of cytokine secretion.

### HIV-1 Challenge of CXCR4 and CCR5 siRNA Transgenic Macrophages

To determine if the stable down regulation of CXCR4 and CCR5 conferred resistance to HIV-1 infection in CD34+ derived macrophages, cells were challenged with X4 (NL4-3) or R5 (BaL-1) tropic strains of HIV-1. Both NL4-3 and BaL-1 challenge experiments were carried out at an m.o.i. of 0.01 for 2 hours in the presence of polybrene (4 ug/ml). Viral supernatants were collected on various days post-infection for p24 antigen ELISAs. To quantify viral p24 levels, a Coulter-p24 kit (Beckman Coulter, Fullerton, CA) was used.

## Competing interests

The author(s) declare that they have no competing interests.

## Authors' contributions

JA performed all experiments. RA was responsible for the overall experimental design and implementation of the project.
